# Mitigating Environmental Impact Through the Use of Rice Husk Ash in Sustainable Concrete: Experimental Study, Numerical Modelling, and Optimisation

**DOI:** 10.3390/ma18143298

**Published:** 2025-07-13

**Authors:** Md Jihad Miah, Mohammad Shamim Miah, Humera Mughal, Noor Md. Sadiqul Hasan

**Affiliations:** 1 Civil Engineering, School of Architecture, Technology and Engineering, University of Brighton, Lewes Road, Brighton BN2 4GJ, UK; 2 Institute of Engineering Geodesy and Measurement Systems, Graz University of Technology, 8010 Graz, Austria; miah@tugraz.at; 3 College of Architecture and Design, Prince Sultan University, Salman Neighborhood, Riyadh 12435, Saudi Arabia; hmughal@psu.edu.sa; 4 Department of Civil Engineering, International University of Business Agriculture and Technology, Dhaka 1230, Bangladesh; nmshasan@iubat.edu

**Keywords:** rice husk ash, concrete, stress–strain profile, strength, data-based model, optimisation

## Abstract

Cement production significantly contributes to CO_2_ emissions (8% of worldwide CO_2_ emissions) and global warming, accelerating climate change and increasing air pollution, which harms ecosystems and human health. To this end, this research investigates the fresh and hardened properties of sustainable concrete fabricated with three different replacement percentages (0%, 5%, and 10% by weight) of ordinary Portland cement (OPC) using rice husk ash (RHA). The hardened properties were evaluated at 14, 28, 60, 90, and 120 days of water curing. In addition, data-based models were developed, validated, and optimised, and the models were compared with experimental results and validated with the literature findings. The outcomes reveal that the slump values increased (17% higher) with the increased content of RHA, which aligns with the lower temperatures (12% lower) of freshly mixed concrete with RHA than the control mix (100% OPC). The slopes of the stress–strain profiles decreased at early ages and improved at longer curing ages (more than 28 days), especially for mixes with 5% RHA. The compressive strength decreased slightly (18% at 28 days) with increased percentages of RHA, which was minimised with increased curing ages (8% at 90 days). The data-based model accurately predicted the stress–strain profiles (coefficient of determination, $R^{2}$ ≈ 0.9950–0.9993) and compressive strength at each curing age, including crack progression (i.e., highly nonlinear region) and validates its effectiveness. In contrast, the optimisation model shows excellent results, mirroring the experimental data throughout the profile. These outcomes indicate that the 10% RHA could potentially replace OPC due to its lower reduction in strength (8% at 90 days), which in turn lowers CO_2_ emissions and promotes sustainability.

## 1. Introduction

Global cement consumption continues to rise at an unprecedented rate, driven by expanding urbanisation, large-scale infrastructure projects, and booming construction activities across both developing and developed nations [1,2]. As the fundamental component of concrete, cement serves as the essential building block for modern architecture, transportation networks, and urban development [3,4,5]. However, this essential material comes with a significant environmental cost—the cement industry generates nearly 8% of worldwide carbon dioxide (CO_2_) emissions, primarily due to energy-intensive production methods and chemical reactions inherent in its manufacturing [6,7]. Reducing cement-related emissions without compromising construction demands requires innovative approaches. These include the development of alternative binders, the incorporation of supplementary cementitious materials, and the implementation of carbon capture technologies. Additional strategies involve using alternative fuels and adopting advanced manufacturing processes [8,9,10,11].

Supplementary cementitious materials (SCMs), such as slag, fly ash, and silica fume, are increasingly valued in modern construction for enhancing performance while reducing their environmental footprint [11,12,13]. These industrial byproducts improve workability, long-term strength, and durability by filling microvoids and reacting with calcium hydroxide to form additional binding phases [11,12,13,14,15,16,17,18]. Environmentally, SCMs offer triple benefits: they divert industrial waste from landfills, reduce the clinker factor in cement production (thereby lowering CO_2_ emissions), and decrease the energy-intensive processing of raw materials [9,10,11,12,13,14]. Researchers have found that using up to 30% fly ash or slag enhances the mechanical strength and durability of concrete [11]. However, exceeding 30% fly ash or slag can lead to a reduction in concrete strength [11,19,20].

However, conventional SCMs, such as slag, fly ash, and silica fume, are limited due to supply issues and industrial changes [10,21]. With rising cement demand and increasingly stringent decarbonization targets, exploring alternative materials that can reliably support sustainable construction is crucial. Rice husk ash (RHA), an agricultural byproduct derived from the burning of rice husks, has emerged as a promising sustainable alternative to traditional cement in concrete [22,23]. With a high silica content (85–95%) in an amorphous form, RHA exhibits strong pozzolanic activity, enhancing the long-term strength and durability of concrete by reacting with calcium hydroxide to form additional binding compounds [22,23,24]. Its fine particles improve particle packing density, reducing permeability and increasing resistance to chemical attack. Experimental findings demonstrate that ultra-high-performance concrete containing RHA particles (3.6–9 µm) can attain compressive strengths exceeding 150 MPa under standard curing conditions [25]. Notably, RHA contributes more significantly to strength enhancement than silica fume (SF). A ternary cement mixture with 10% RHA and 10% SF outperformed the control mix, achieving the highest compressive strength [25]. Sensale [26] stated that the long-term testing revealed that RHA-blended concrete achieved superior 91-day compressive strength compared to control concrete, despite showing variable early-age (7–28 days) performance between the two RHA types studied. It was concluded that the strength enhancement primarily resulted from particle packing and microstructure densification (filler effect) rather than chemical reactions [26]. In contrast, concrete incorporating controlled-incineration RHA demonstrated strength gains predominantly attributable to pozzolanic activity [26,27,28].

RHA shows excellent potential as a sustainable alternative to cement in concrete. However, if untreated RHA is improperly disposed of, it can create environmental hazards. One common disposal method is the open-field burning of rice husks, which releases fine and toxic particulate matter, carbon monoxide, and other greenhouse gases, thereby worsening air pollution and posing respiratory health risks [22,23,24]. Additionally, uncontrolled dumping of RHA can lead to spikes in soil alkalinity and silica leaching, which disrupt ecosystems and contaminate groundwater [22]. Large stockpiles of RHA also pose a risk of spontaneous combustion, thereby contributing to increased greenhouse gas emissions. While RHA can be a valuable substitute for cement, its unregulated production and disposal can exacerbate environmental degradation [22,23,24]. This situation underscores the need for stricter waste management policies and cleaner processing techniques, such as controlled pyrolysis, to minimise harm. Processing RHA through controlled combustion at temperatures between 500 and 700 °C transforms it into a reactive pozzolan, which can reduce the carbon footprint of concrete by replacing cement [22,23,24,25,26]. This approach not only diverts agricultural waste from landfills but also enhances the strength and durability of concrete, providing a dual solution for both waste management and sustainable construction. Therefore, implementing proper regulations for RHA production and application is essential to mitigate its environmental risks while maximising its ecological benefits.

While RHA offers better mechanical and durability properties, as well as environmental advantages, by utilising agricultural waste and reducing cement demand, several researchers have reported that its incorporation has several limitations, often leading to early-age and even later-age strength reduction in concrete [29,30,31,32,33,34,35,36]. This reduction primarily results from the slower pozzolanic reactivity of RHA compared to Portland cement, resulting in delayed strength development at 7–28 days [29,30,31,32,33,34,35,36]. The extent of strength reduction depends on multiple factors, including RHA’s particle size, silica crystallinity, and replacement percentage. Finely ground amorphous RHA with high reactivity can eventually match or exceed the strength of control concrete through prolonged pozzolanic reactions, typically beyond 28 days. However, unprocessed or poorly burnt RHA with crystalline silica content may cause permanent strength deficits due to inadequate chemical contribution [29,30,31,32,33,34,35,36,37]. Additionally, RHA’s porous structure may increase water demand and shrinkage, potentially impacting long-term crack resistance and structural integrity. These factors necessitate careful optimisation for reliable structural use [29,30,31,32,33,34,35,36,37]. The optimal balance between sustainability goals and structural requirements remains a key research challenge in RHA–concrete applications, particularly for projects demanding early strength development.

Numerical modelling has emerged as a powerful tool for predicting the structural behaviour of concrete, offering precise simulations of load-deflection relationships and compressive strength without the need for extensive experimental trials [38,39]. These models can replicate complex material interactions by leveraging computational methods, such as finite element analysis, machine learning algorithms, or data-driven models, which account for variables like mix composition, curing conditions, and loading scenarios [38,39,40,41]. This predictive capability significantly reduces the need for physical testing, minimising material waste, laboratory costs, and time-intensive iterations. From a sustainability perspective, numerical modelling optimises resource efficiency by enabling virtual prototyping—allowing engineers to refine mixed designs digitally before production [38,39,40,41]. This approach conserves raw materials and lowers the carbon footprint associated with trial batches and failed experiments [38,41]. By accurately forecasting mechanical performance, computational tools support the development of high-performance, low-emission concrete mixes, aligning with circular economy principles. As the construction industry seeks greener alternatives, numerical modelling stands out as a cost-effective, time-saving, and environmentally conscious solution for concrete design and innovation.

Despite its potential as a sustainable cement substitute, RHA concrete requires further study to address critical inconsistencies in the literature. Many studies focus only on short-term strength, while research lacks comprehensive data on long-term strength (up to 120 days). Additionally, the numerical modelling of the RHA concrete, including stress–strain profiles and strength development, is not well known in the literature, whereas data-based mathematical modelling is almost unknown. Bridging these gaps through systematic research is crucial for developing a reliable mix design and practical guidelines for real-world applications. In contrast, if not properly managed or utilised, RHA can seriously threaten the environment, especially in Asian countries where rice is the main food and produces enormous amounts of RHA. To this aim, this study investigated the stress–strain profiles and strength development of concrete mixes containing three different percentages (0%, 5%, and 10% by weight of OPC) of RHA as a replacement of OPC at 14, 28, 60, 90, and 120 days. The selection of RHA content, ranging from 0% to 10%, was made based on a comprehensive literature review and its findings [29,30,33,34,35,37]. While most researchers have explored RHA percentages between 0% and 30% [30,34,35], others have examined ranges of 0% to 15% [29,37] and 0% to 20% of RHA [33]. It has been shown that the strength properties of the concrete decrease as the percentage of RHA increases [29,30,33,34,35,37]. To optimise the effects of RHA on the concrete’s properties, this study focuses on using RHA as a cement replacement at levels ranging from 0% to 10%. A data-driven predictive model was developed, validated, and optimised with experimental results, providing a robust analytical framework for future applications to the concrete industry. Additionally, the model was validated using results from the literature.

## 2. Experimental Methodology

**Figure 1:**
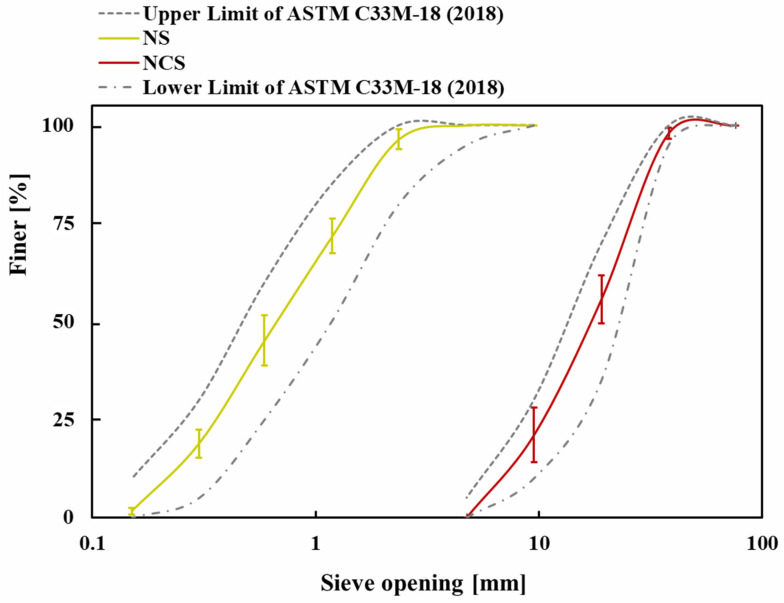
Grain size distribution of NCS and NS compared with the upper and lower limits [43].

**Figure 2:**
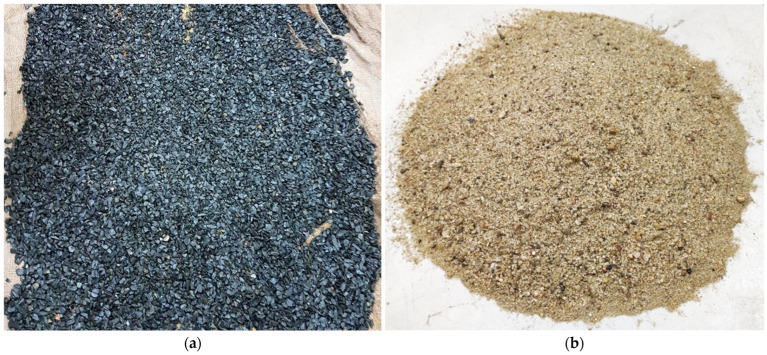

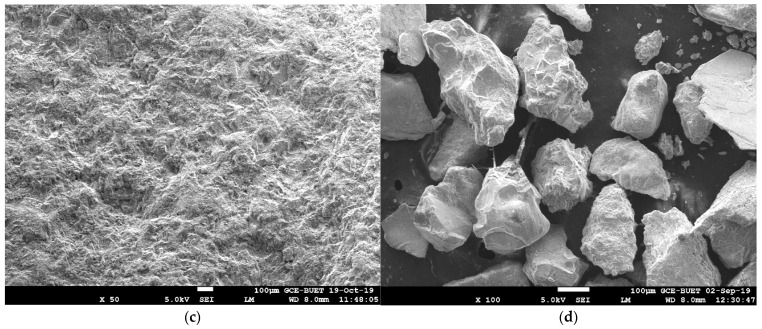
The original image of natural crushed stone–NCS (**a**), natural sand–NS (**b**), SEM image of NCS (**c**), and NS (**d**).

The concrete specimens were prepared using a binder (CEM I 42.5 N), water, natural crushed stone (NCS) as coarse aggregate, and natural sand (NS) as fine aggregate. The aggregates were sieved in accordance with ASTM C136 [42], and their particle size distribution was determined in accordance with ASTM C33 [43], as illustrated in Figure 1. Table 1 summarises the physical properties of the aggregates, as tested per ASTM standards. The coarse aggregate exhibited a specific gravity of 2.73, a unit weight of 1550 kg/m^3^, and an absorption capacity of 1%, while the fine aggregate had values of 2.54, 1530 kg/m^3^, and 3.10%, respectively. Additionally, the Los Angeles abrasion resistance of the coarse aggregate was 12%. The fineness modulus for coarse and fine aggregates was 7.43 and 2.82, respectively. The original images of NCA and NS are illustrated in Figure 2a,b. The maximum and minimum sizes of NCA shown in Figure 2a were 19.05 mm and 4.75 mm, respectively. In contrast, the maximum and minimum sizes of NS illustrated in Figure 2b were 4.75 mm and 0.15 mm, respectively. SEM analysis was conducted to examine the microstructure of the NS and NCA, as shown in Figure 2c,d. The surface texture and internal structure of the aggregates have a significant influence on the strength and durability of the concrete. The SEM images revealed that both NCA and NS possess angular shapes and rough surfaces, enhancing their bonding with the cement mortar. This improved interfacial transition zone (ITZ) minimises bond failure around the aggregates, allowing them to contribute effectively to load-bearing. As a result, the concrete achieved the desired target strength, exceeding 53 MPa at 28 days.

**Figure 3:**
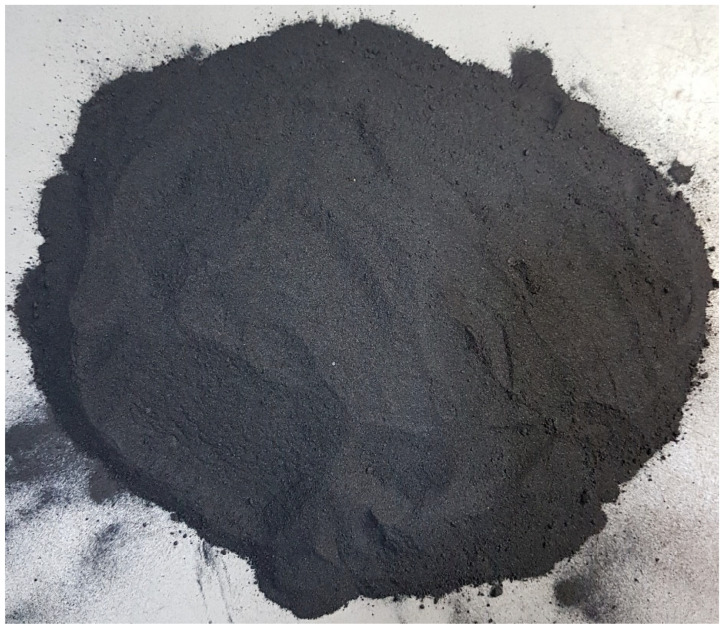
Original image of rice husk ash (RHA).

Ordinary Portland cement (OPC) was used as a binder, sourced from a nearby cement manufacturer. The chemical compositions of OPC were analysed [11] using X-ray fluorescence (XRF), with the findings detailed in Table 2. RHA was produced by combusting rice husks, an agricultural byproduct obtained during the milling of rice. To ensure high quality, fresh husks were collected immediately after milling to minimise contamination from soil or moisture. The rice husks were then combusted under controlled conditions at temperatures between 400 °C and 600 °C for more than two hours. This process is crucial for developing pozzolanic activity. After combustion, the resulting ashes were ground into a fine powder. The ashes are then sieved through a mesh with an opening size of 65 µm to enhance their surface area and reactivity when used as a substitute for cement. Properly processed, RHA becomes a highly reactive material suitable for partial cement replacement in concrete, which dramatically influences the properties of concrete. The image of RHA is illustrated in Figure 3. The chemical composition of RHA data was studied in [44] and presented in Table 2. The analysis revealed that RHA contains substantially less CaO compared to OPC, suggesting a slower hydration rate. Conversely, RHA exhibited higher concentrations of SiO_2_ than OPC. Additionally, Table 2 includes the chemical composition of NCS and NS. The specific gravity of OPC and RHA is about 3.10 and 2.3 [22].

Three concrete mixtures were prepared with a constant water-to-binder (w/b) ratio of 0.35. Two of these mixtures incorporated RHA as partial replacements for OPC at 5% and 10% by weight of the OPC. These were labelled 5% RHA and 10% RHA, respectively, while the control mix contained 100% OPC. To ensure a low w/b ratio and minimise air voids, a superplasticiser was added at 0.5% by weight of the binder to enhance the workability of the freshly mixed concrete. Table 3 summarises the mix proportions. The workability of fresh concrete was evaluated using slump tests, and the temperature of each mixture was recorded with a digital thermometer to monitor the development of hydration heat at different replacement levels.

The mechanical properties of all concrete mixtures were assessed through compressive strength tests using cylindrical specimens with dimensions of 100 mm in diameter and 200 mm in height. Each mould was filled with fresh concrete in three equal layers, with each layer receiving 25 compaction strokes. After casting, the specimens were left in the laboratory for 24 h and covered with plastic sheeting to minimise moisture loss. Once demoulded, the samples underwent water curing at a temperature of 20 ± 2 °C until testing, which occurred at 14, 28, 60, 90, and 120 days. Strength testing was conducted in accordance with ASTM C39 [45] guidelines for evaluating compression. A specialised strain measurement system, which included three digital dial gauges and an extensometer with a 100 mm gauge length positioned at mid-height, recorded deformation during the compression tests of concrete. Three specimens were tested for their strength properties at each mixture and curing period, and the results were reported as the average of these replicates. The error bars in the compressive strength results illustrated the data range from the three tested cylinders, as discussed in Section 4.2. After the tests, a thorough examination of fracture patterns on the failed specimens was conducted.

## 3. Data-Based Modelling and Optimisation

In recent decades, the development and progress of sensor technologies have led to their increased adoption and use across nearly all fields of science and engineering. In this context, civil engineering also incorporates sensors (e.g., laser triangulation sensors, LVDT-type sensors, accelerometers) for diverse applications, such as data measurement and monitoring [46]. The integration of multiple sensors has made data utilisation and management a growing trend in civil engineering and infrastructure. Sensor data is closely connected to monitoring applications, which can be either real-time or offline. When monitoring and assessment tasks are required, an initial model is essential to evaluate and predict changes and potential outcomes. In this regard, data-driven mathematical models can be crucial for characterising existing data and forecasting future scenarios. Data-driven models can significantly help in avoiding unnecessary experiments, saving both time and money. A well-developed data-based mathematical model can explain existing data phenomena and predict unseen scenarios with a certain level of confidence [46].

Choosing a type of model can be a difficult task, as it is often a highly individual problem linked to the original measured data. In reality, many models exist; some notable examples include linear regression, simple regression, the general linear model, polynomial regression, vector generalised linear model, logistic regression, nonlinear regression, and artificial intelligence models [47,48,49,50]. Regression models are applicable across various fields of science and engineering because of their simplicity and practicality. For example, supervised machine learning (ML) algorithms are part of the regression model family [49]. As previously mentioned, selecting a mathematical model depends on the specific problem; in this case, a polynomial regression type has been chosen to characterise experimentally measured data. These models are selected due to their excellent performance, and they are generally straightforward to manage [38,46,50]. The mathematical expression of the developed model in matrix-vector form is provided in Equations (1) and (2).

(1) $$\sigma^{stress}=\alpha_{1}\varepsilon^{4}+\alpha_{2}\varepsilon^{3}+\alpha_{3}\varepsilon^{2}+\alpha_{4}\varepsilon +\alpha_{5}$$

(2) $$\sigma^{stress}=\underset{\alpha }{\underbrace{\begin{array}{ccccc} {}[\alpha_{1} & \alpha_{2} & \alpha_{3} & \alpha_{4} & \alpha_{5}] \end{array}}}\left\{\begin{array}{c} \varepsilon^{4}\\ \begin{array}{c} \varepsilon^{3}\\ \varepsilon^{2} \end{array}\\ \varepsilon \\ 1 \end{array}\right\}$$

where $\sigma^{stress}$ is the output, e.g., stress, $\alpha_{1}$, $\alpha_{2}$, $\alpha_{3}$, $\alpha_{4}$, and $\alpha_{5}$, are the unknown coefficients of the model, ε is the main input variable, e.g., strain. It needs to be remembered that the coefficients (e.g., $\alpha_{1}$, $\alpha_{2}$, $\alpha_{3}$, $\alpha_{4}$, and $\alpha_{5}$) are tunable; hence, they are not the same for all models. The aforementioned Equations (1) and (2) can be expressed as below (Equation (3)) with the sample coefficients.

(3) $$\sigma^{stress}=\begin{array}{ccccc} {}[{-2.37e}^{12} & {1.17e}^{10} & -{2.48e}^{7} & -{4.33e}^{4} & -1.91] \end{array}\left\{\begin{array}{c} \varepsilon^{4}\\ \begin{array}{c} \varepsilon^{3}\\ \varepsilon^{2} \end{array}\\ \varepsilon \\ 1 \end{array}\right\}$$

The coefficient of determination (CoD) is an estimation based on the model outputs that measures how closely the observed behaviour is rendered by the developed model [51,52]. Typically, the CoD is given by $R^{2}$, and the use of $R^{2}$ can be explained such that a higher value of $R^{2}$ tells that the accuracy of the representative model is higher, and vice versa. In this study, the CoD has been estimated for all of the developed models. The mathematical expression of $R^{2}$ is given in Equations (4) and (5) [51,52].

(4) $$R^{2}=1-\frac{{SS}^{residual}}{{SS}^{total}}$$

The previous Equation of $R^{2}$ can be rearranged by inserting the expressions of the residual sum of squares (${SS}^{residual}$) and the total sum of squares (${SS}^{total}$) as follows:

(5) $$R^{2}=1-\frac{\sum_{i}{\left(y_{i}-f_{i}\right)}^{2}}{\sum_{i}{\left(y_{i}-\bar{y}\right)}^{2}}$$

Finally, the mean value (, $\bar{y}$) of the data is described by Equation (6),

(6) $$\bar{y}=\frac{1}{N}\sum_{i=1}^{N}\left(y_{i}\right)$$

where ${SS}^{residual}$ is the residual sum of squares, ${SS}^{total}$ is the total sum of squares, i represents the actual data, N is the length of the data, $\bar{y}$ is the mean of the used data sample.

After developing mathematical models, their performance can be further enhanced through optimisation [53]. The optimisation technique is an advanced method to improve the performance of any model. Many optimisation techniques are available, and designers must carefully choose the appropriate optimisation algorithms based on individual problems. For instance, if a proper optimisation algorithm is not chosen, the optimisation may only settle at the local minima instead of the global minimum. As a result, the problem will not be fully optimised. Therefore, designers must be careful when choosing between linear and nonlinear, as well as constrained and unconstrained algorithms [54,55,56]. The required computing power is also an important factor in selecting an appropriate optimisation algorithm. In this context, Ref. [57] has compared the performance of Genetic Algorithm (GA), Particle Swarm Optimisation (PSO), and Constrained Nonlinear Optimisation (CNO) Algorithm. The outcome of the earlier-mentioned study demonstrates that significant computing power is required for both GA and PSO, whereas CNO exhibits markedly better performance and demands considerably less computing power and simulation time. Therefore, a nonlinear derivative-free optimisation algorithm, also known as a heuristic or search-based algorithm, is utilised to carry out the optimisation. For this purpose, the Nelder–Mead Simplex (NMS) Method is employed due to its excellent performance and its lower requirement for computational power and simulation time [58,59,60,61,62]. It was mentioned earlier that selecting an optimisation algorithm that avoids getting stuck in local minima is important; instead, it should search for the global minimum [55,58,61,62]. To perform any optimisation, the user must define an objective function based on the individual’s problem [57,59,61,62]. Since optimisation algorithms aim to minimise the error of the specified objective or cost function, performance is improved. The objective function for the investigated problem is given in Equation (7).

(7) $$J^{opt}=\sqrt{\sum_{i=1}^{N}\frac{{\left|f^{true}-f^{model}\right|}^{2}}{{\left|f^{true}\right|}^{2}}}$$

where $f^{true}$ is the experimentally measured true data, $f^{model}$ is the estimated output of the model, $J^{opt}$ is known as the objective or cost function.

Interested readers may consult the following articles to learn more about optimisation in general [54,55,56,58,61,62]. Due to the inherent complexity of modelling and optimisation, all analyses were carried out using MATLAB^®^ R2020a. However, the confidence levels were estimated based on the standard deviation in this study, with a 95% confidence interval.

## 4. Experimental Results and Discussion

### 4.1. Fresh Properties of Concrete Mixes

**Figure 4:**
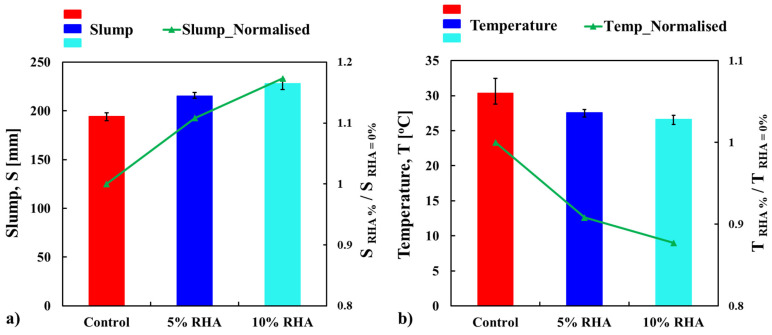
Average slump (**a**) and temperature (**b**) test values of freshly mixed concrete mixes.

The workability of the concrete mixes was assessed by measuring the slump values of concrete containing three different percentages of RHA—0%, 5%, and 10% by weight of ordinary OPC. To finalise the mix design, trial mixes were cast without a superplasticiser, which led to a reduction in workability. To achieve a low water-to-binder ratio (w/b) of 0.35, minimise air voids, and improve workability, a superplasticiser was added at a ratio of 0.5% by weight of the binder. The results from the trial mixes were not recorded or presented. Figure 4a displays the average workability and normalised results for three concrete mixes with varying percentages of RHA and superplasticiser. The error bars represent the data range from the three-slump tests of each freshly mixed concrete. The addition of RHA significantly influences the workability of fresh concrete, with higher amounts leading to greater improvements. Specifically, the mixes with 5% and 10% RHA showed slump values that were 11% and 17% higher, respectively, than the control mix, as shown in Figure 4a. This suggests that RHA can decrease the water demand by working together with the superplasticiser, thereby enhancing the handling and placement of fresh concrete.

**Figure 5:**
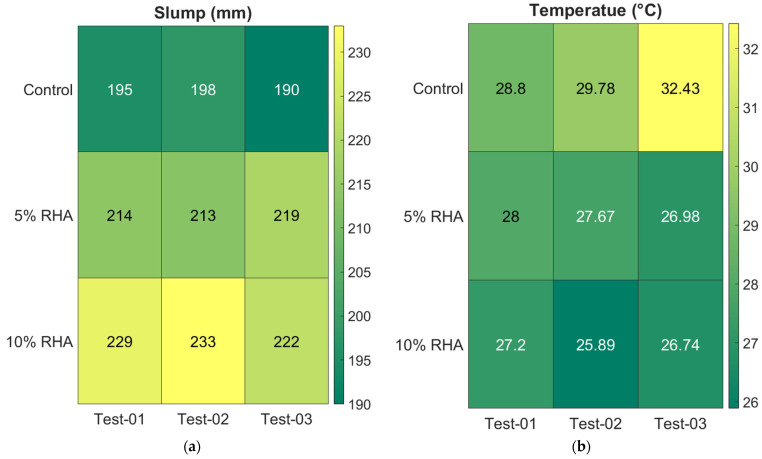
Colour illustration of the individual slump (**a**) and temperature (**b**) test values of freshly mixed concrete mixes.

Figure 5a was generated using colour images to better illustrate the test results. The gradient from yellow to green indicates the maximum and minimum slump values recorded in each mix’s three tests. The highest slump value of 233 mm was recorded for the mix with 10% RHA in test 02, while the control mix registered the lowest slump value of 190 mm in test 03. Overall, these colour intensity results are engaging and help readers better grasp the data. It has been observed that better repeatability and consistency were found in the concrete mix with 5% RHA. In contrast, moderate variability was reported in the control mix, and more variable data were observed in the concrete mix with 10% RHA. The standard deviations of the concrete mixes containing 0, 5, and 10% RHA are 4.04, 3.21, and 5.57, respectively.

The temperature of freshly mixed concrete at the time of placement can influence its slump characteristics. The temperatures of the concrete mixes were measured using a digital concrete thermometer. Compared to the mixes containing rice husk ash (RHA), the control mix (100% Ordinary Portland Cement, OPC) showed a higher temperature, as depicted in Figure 4b. The addition of RHA led to a reduction in the temperature of fresh concrete, which is attributed to its lower heat of hydration compared to the control mix. Specifically, the temperature dropped by 9% and 12% in the mixes with 5% and 10% RHA, respectively, relative to the control mix. Figure 5b displays colour images illustrating the concrete temperatures for each test. The colour images showed consistent trends. The highest temperature of 32.43 °C was recorded for the control mix during test 03, while the mix with 10% RHA registered a minimum temperature of 25.89 °C in test 02. Overall, these colour-intensity results offer valuable insights and help readers better understand the temperature data. The measured temperatures showed that the concrete mix containing 5% RHA had higher repeatability and more consistent results compared to other mixes. The mix with 10% RHA experienced moderate fluctuations, while the control mix demonstrated the greatest variability. The calculated standard deviations for mixes with 0%, 5%, and 10% RHA are 1.88, 0.52, and 0.66, respectively.

As previously reported, the trial mix without superplasticiser showed a significant decrease in workability. However, once the superplasticiser was introduced, the workability of the mixes containing RHA greatly improved. The enhancement in slump value can be attributed to the mechanisms of the superplasticiser, which disperses cement particles through electrostatic repulsion and steric hindrance [3,63,64]. These high-range water reducers adsorb onto the surface of cement grains, imparting a negative charge that causes the particles to repel each other, breaking up flocculation and releasing trapped water [3,63,64]. This dispersion reduces internal friction, enabling the mixture to flow more easily without requiring additional water. Moreover, the improvement in workability may also result from the fine particle size and pozzolanic properties of RHA. The finer particles of RHA fill the voids between cement grains, reducing internal friction and shear resistance while improving particle packing density. This results in a smoother, more cohesive mixture with lower water requirements for achieving a specific slump. As illustrated in Figure 2a,d, the stone aggregate exhibits significantly higher angularity and surface roughness. Since RHA has a lower specific gravity—approximately 3.10 for OPC and 2.3 for RHA—the proportion of RHA used in the mix is greater when measured by weight replacement. This increased amount, combined with the fine particle size and uniform particle distribution of RHA, enables it to coat the crushed aggregates more effectively, providing additional binder compared to the control mix. The enhanced dispersion and well-coated aggregates from RHA particles minimise internal frictional stress among the crushed aggregates, promoting better flowability and workability [3,11]. This increased slump improves the concrete’s pumpability and ease of compaction, resulting in a denser matrix with fewer voids.

The present study indicates that concrete mixed with RHA maintains lower temperatures than control concrete in its freshly mixed state, as illustrated in Figure 4b and Figure 5b. This temperature difference can significantly impact the workability of the concrete. The lower temperature associated with RHA can slow the hydration process, delaying the formation of rigid cementitious gels that typically reduce fluidity. This extended dormant phase allows the mixture to remain in a more fluid state for a longer period, thus improving slump retention. As shown in Table 2, the CaO content in RHA is significantly lower than that in OPC (0.21% for RHA compared to 65.01% for OPC). This lower CaO content reduces the exothermic reaction with water, resulting in less heat generation. Consequently, early cement hydration is slowed, which lowers the overall temperature of the mixture, particularly as RHA content increases. Additionally, since viscosity is temperature-dependent, the lower temperature of the RHA mixture reduces internal friction within the paste. This reduction in friction facilitates improved particle mobility and easier consolidation. Together, the effects of delayed setting, reduced water loss, and decreased paste viscosity contribute to prolonged workability. This makes concrete easier to handle, place, and finish, which is especially beneficial in large-scale or high-performance project applications. Furthermore, the reduced heat generation mitigates thermal stress and cracking, suggesting that RHA effectively prevents thermal issues in mass concrete applications. In contrast, the higher temperature of the control mix accelerates cement hydration, which may result in decreased workability over time.

Compared to slag and fly ash, the workability of RHA shows similar behaviour to concrete mixes with slag or fly ash, as both cases exhibit increased workability with higher contents of RHA, slag, or fly ash [11,17]. For example, the workability measurements indicated a direct relationship between fly ash (FA) content and concrete slump, with mixes containing 50%, 70%, and 90% FA demonstrating 33%, 39%, and 48% higher slump values, respectively, compared to control concrete (100% OPC concrete) [17]. Likewise, the addition of ground granulated blast furnace slag (GGBS) at 20%, 40%, and 60% replacement levels improved workability, resulting in slump increases of 10%, 70%, and 100% over the control mix [14,18], where the slump of the studied concrete mixes increased by 11% and 17% in the mixes with RHA compared to the control concrete, which aligns with the literature [11,14,17,18].

### 4.2. Mechanical Properties

#### 4.2.1. Stress–Strain Profile of Concrete Mixes

**Figure 6:**
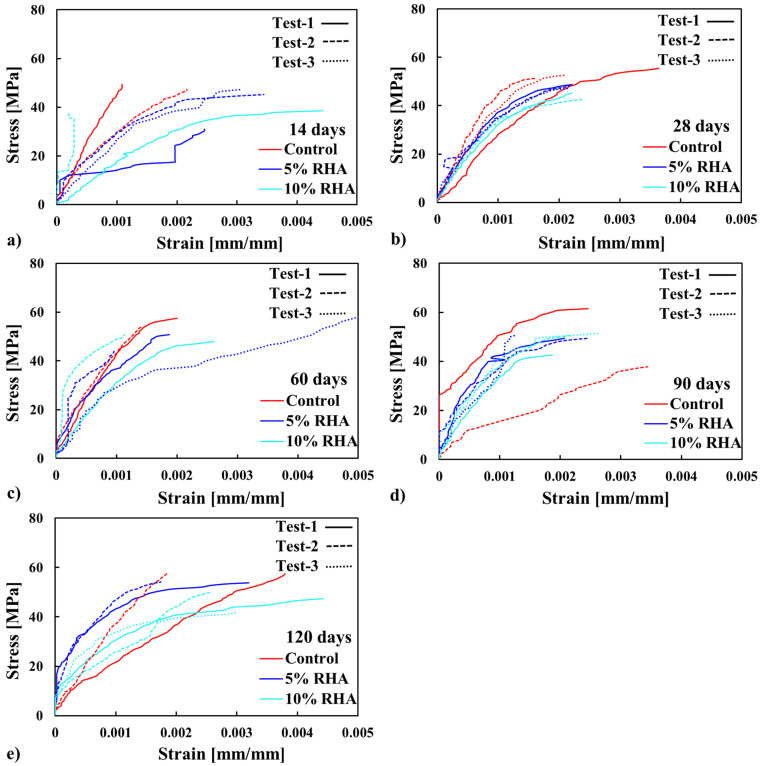
Stress–strain profiles of concrete with varying RHA contents at different curing ages: (**a**) 14 days, (**b**) 28 days, (**c**) 60 days, (**d**) 90 days, and (**e**) 120 days.

The uniaxial compressive stress–strain response of each concrete mix was assessed using three cylindrical specimens measuring 100 × 200 mm at 14, 28, 60, 90, and 120 days of curing. In most cases, the resulting stress–strain curves, illustrated in Figure 6, exhibit a distinct linear region before reaching peak stress, consistent with findings in existing research [31,36,65,66]. However, variations are noticeable in the steepness of this initial ascending phase, which relates to the elastic modulus. As shown in Figure 6, the stress–strain behaviour of concrete mixes with RHA displays distinct differences due to variations in their microstructural composition. In most RHA concrete specimens, the curve initially exhibits a linear elastic response, followed by gradual nonlinearity as microcracks develop in the mortar matrix. After reaching peak stress, RHA concrete experiences significant strain softening, characterised by a steep decline in strength caused by unstable crack propagation and weak interfacial transition zones (ITZs) between aggregates and binder paste. In contrast, the control concrete has a steeper elastic slope, reflecting its higher stiffness and maintaining linearity up to a higher stress level due to its denser microstructure and stronger ITZs.

Post-peak behaviour in control concrete is more brittle, with a rapid stress drop caused by the sudden failure of tightly bonded constituents. In contrast, RHA concrete mixes exhibit relatively higher ductility (especially at the 5% RHA mix at 60 days), owing to their ability to redistribute stresses through gradual microcracking. The increased strength and lower porosity in control concrete limit energy dissipation mechanisms, making it more prone to sudden failure. In contrast, RHA mixes with looser structures permit greater inelastic deformation before collapse. These differences emphasise how RHA in the mix affects crack development and failure modes in concrete structures.

As the age of curing increases, the slope of the stress–strain curves, which represent the elastic modulus, eventually exceeds that of the control mix, especially at 5% RHA mix concrete. This behaviour can be attributed to the increased formation of primary calcium silicate hydrate (C-S-H) gels over time, as the cement further reacts. The finer particles of RHA could fill microvoids, refining the pore structure and strengthening the ITZs. Moreover, RHA enhances pozzolanic reactions, where the silica in the RHA reacts with calcium hydroxide (Ca(OH)_2_) to produce an additional C-S-H gel (i.e., Ca(OH)_2_ + RHA + H_2_O = C-S-H) [31,36,65,66]. As a result, it densifies the matrix and enhances deformation resistance by limiting microcrack development, delaying localised failure, and allowing for better stress redistribution, particularly in the 5% RHA mix. However, at higher replacement levels, such as 10%, excessive RHA can lead to increased porosity due to its relatively unreactive nature and lower cement content, ultimately reducing density and stiffness. Moreover, in the post-peak region, control samples exhibit a rapid loss of load-bearing capacity, whereas concretes incorporating RHA show a more gradual decline. This decline becomes even less pronounced with higher RHA content, indicating slightly improved toughness and reduced brittleness in the modified concretes containing RHA. The stress–strain responses of several specimens, as shown in Figure 6, were inconsistent due to poor stress distribution, leading to premature failures. Overall, while the slope of the curves for the mix containing RHA decreased at early ages, it improved later. Also, the slower decline in stress–strain response demonstrates RHA’s ability to mitigate brittleness by improving fracture toughness, whereas the control mix fails abruptly due to unhindered crack growth. Thus, RHA incorporation alters the failure mechanism by promoting a more ductile, energy-absorbing fracture pattern than conventional concrete, although further study is needed [31,36].

#### 4.2.2. Compressive Strength of Concrete Mixes

**Figure 7:**
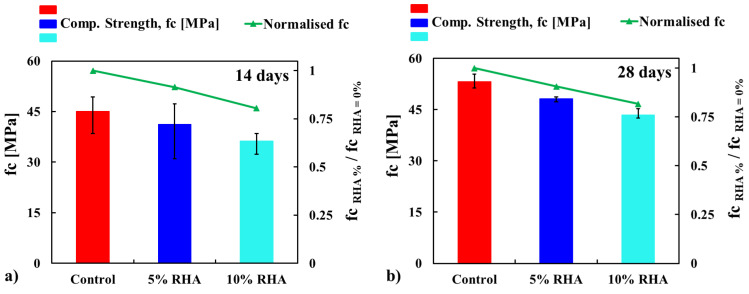

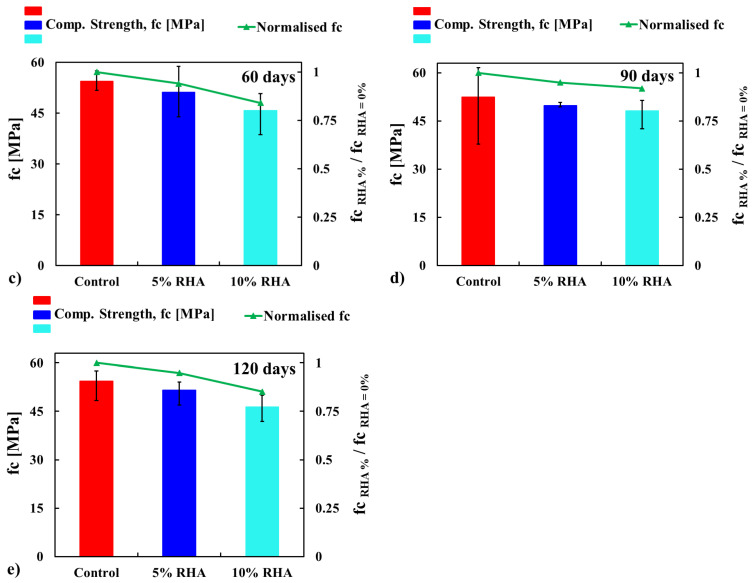
Compressive strength (fc) of concrete with different contents of RHA at different curing ages: (**a**) 14 days, (**b**) 28 days, (**c**) 60 days, (**d**) 90 days, and (**e**) 120 days.

**Figure 8:**
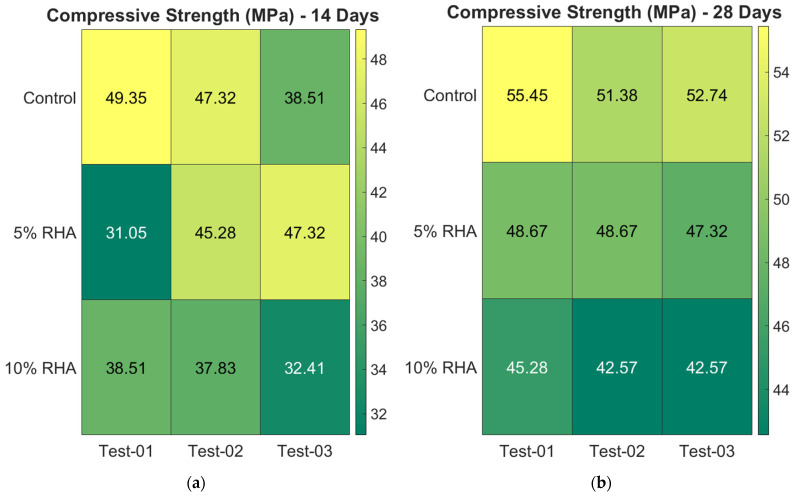

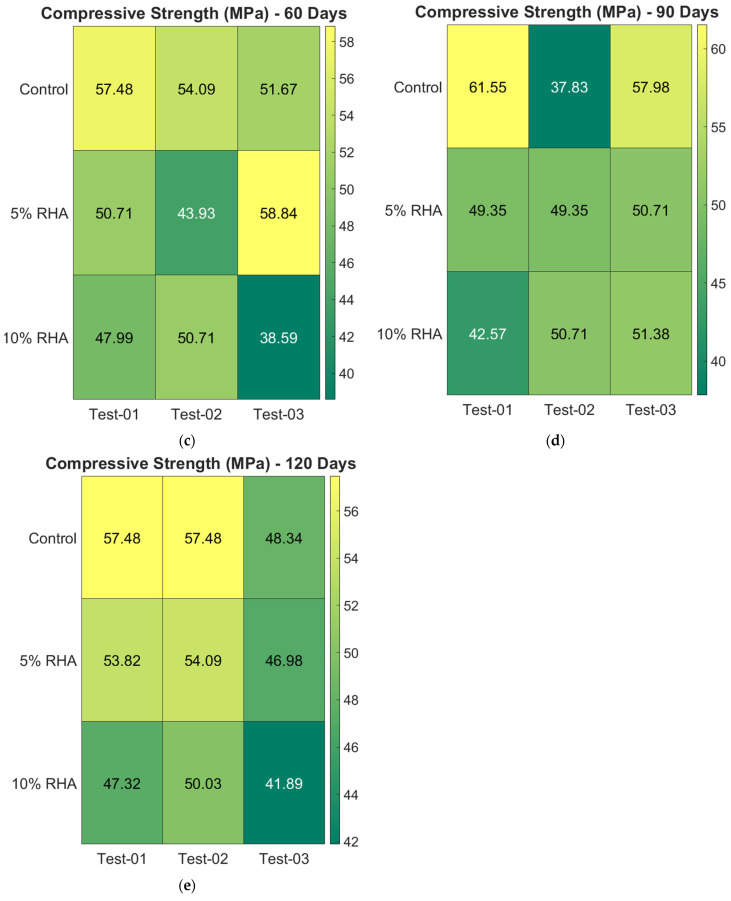
Colour illustration of the individual compressive strength test values of concrete mixes.

The average compressive strength results for concrete mixtures containing RHA at different curing ages—specifically 14, 28, 60, 90, and 120 days—are presented in Figure 7. Three cylindrical specimens were tested for each concrete mix and curing age to evaluate their compressive strength. The strength results are presented as the average of these replicates. In Figure 7, which displays the compressive strength of the tested cylinders, the error bars indicate the range of the data. This figure also includes a line representing the normalised compressive strength (i.e., $f_{c}^{\%RHA}$/$f_{c}^{Control}$) values for the concrete mixes. To enhance the visualisation of the test results for each specimen, Figure 8 employs colour images. The gradient from yellow to green indicates the maximum and minimum compressive strength values recorded from the three tests conducted on each mix.

It has been observed that the compressive strength decreases as the RHA content increases. However, this reduction in strength is minimised with longer curing periods. Most tests showed consistent patterns, as seen in the colour-coded data in Figure 8. The specimens’ strength remained consistent overall; however, several variances in the data were observed, as shown in Figure 8. The most noticeable variability occurred during the 14-day test, with standard deviations of 5.76, 8.86, and 3.34 for the concrete mixes containing 0%, 5%, and 10% RHA, respectively. In comparison, the 28-day test results demonstrated better repeatability, with standard deviations of 2.07, 0.78, and 1.57. Greater variability was seen in the data from strength tests at 60 and 90 days, with standard deviations of 7.47 for the 5% RHA concrete at 60 days and 12.79 for the control concrete at 90 days. At 120 days, the data were more consistent, showing standard deviations of 5.28, 4.03, and 4.14 for the concrete mixes with 0%, 5%, and 10% RHA, respectively. 

In a 14-day test, the compressive strength of concrete mixes containing 5% and 10% RHA was reduced by 8.5% and 19.5%, respectively, compared to the control mix. At this age, concrete with RHA generally shows lower compressive strength than control concrete due to delayed hydration reactions. RHA, a highly porous and reactive pozzolan, partially replaces cement, reducing the immediate availability of C-S-H gels needed for early strength development, which also reduces the production of Ca(OH)_2_ and reacts more slowly through its pozzolanic activity [22,24]. This slower reaction consumes less Ca(OH)_2_ to produce secondary C-S-H gels over time, which is mainly activated in long-term curing. As a result, at 14 days, the microstructure of RHA-modified concrete shows fewer binding phases and increased porosity and permeability due to well-connected pores. Additionally, a weaker ITZ near the aggregates, compared to concrete made with 100% cement (control concrete), causes reduced strength at this early age.

**Figure 9:**
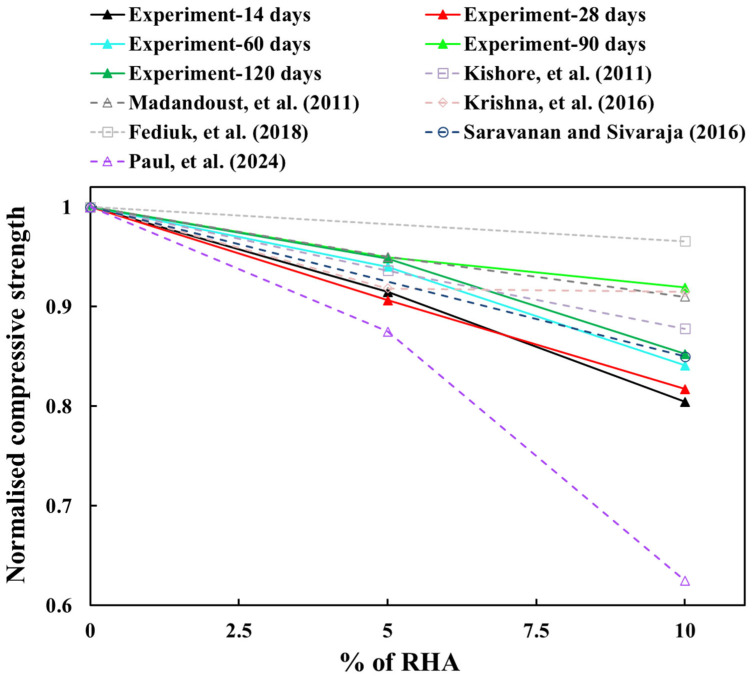
Normalised compressive strength of concrete mixes fabricated with different percentages of RHA tested at different ages and compared with the results at 28 days from the literature [29,30,33,34,35,37].

At 28 days, the strength reduction of the concrete mix was 9% and 18%, respectively, compared to the control mix. A similar reduction in strength was observed at 14 days. Nevertheless, the rate of strength reduction was less significant during the longer curing periods. For example, at 90 days, the strength decreased by only 5% and 8% with 5% RHA and 10% RHA concrete, respectively, in comparison to the control mix. These findings are consistent with the literature findings [29,30,33,34,35,37]. To compare the experimental results of the concrete mix containing RHA, Figure 9 was created using data from the literature [29,30,33,34,35,37]. In Figure 9, the results obtained from the literature at 28 days of water curing are compared with the experimental results across all curing ages. The outcomes from the literature indicate that compressive strength decreased as the content of RHA increased, which aligns well with the experimental results. However, the reduction in strength percentage with increasing curing ages (more than 28 days) can also be clearly observed.

The enhanced strength of concrete that incorporates RHA can be attributed to its micro-filler effect and the additional C-S-H gels generated through pozzolanic reactions [29,30,33,34,35,37]. Due to their fine particle size, RHA effectively fills microscopic voids in the concrete, reducing porosity and permeability. This process produces a denser matrix and a stronger ITZ between the cement paste and the aggregates. This leads to higher compressive strength as failure occurs through both the aggregate and the mortar [3,11]. Over time, the silica-rich particles of RHA react slowly with Ca(OH)_2_, a byproduct of the cement hydration process, to form additional C-S-H gel. This secondary C-S-H gel progressively fills capillary pores, further reducing porosity and permeability. It also strengthens the ITZ between the aggregates and the cement matrix, ultimately enhancing the overall mechanical performance of the concrete. C-S-H gels are the primary phase contributing to the strength of cementitious systems [3,11]. In contrast, control concrete typically achieves much of its strength within 28 days due to primary hydration. This type of concrete relies solely on the hydration of clinker, producing primarily initial C-S-H gels without the occurrence of pozzolanic reactions.

Comparing the mechanical performance with slag and fly ash (FA), the RHA showed poor results, as the strength of concrete mixes increased up to certain percentages of slag or fly ash and then declined at higher replacement levels [11]. Conversely, the strength decreased as RHA content increased, as illustrated in Figure 7, Figure 8 and Figure 9. A study revealed that initial testing at 28 days showed comparable compressive strength between control concrete and modified mixes with up to 30% FA or GGBS [11]. However, subsequent assessments at 60 and 90 days indicated better strength gains (around 7–9% for 30% FA and 13–15% for 30% GGBS) in these blended mixes [11]. The optimised 30% FA and 30% GGBS replacement formulations demonstrated improved durability, exhibiting lower porosity and water absorption compared to conventional concrete [11]. Nevertheless, some research indicated that 30% and 50% FA substitutions resulted in strength reductions of 38% and 47%, respectively [19]. Similar replacements with slag caused more moderate decreases of 13% and 22% [19]. These findings align with the findings for RHA concrete, as shown in Figure 7, Figure 8 and Figure 9. Extended testing at higher replacement levels revealed that incorporating 40%, 55%, and 70% FA resulted in reductions of 17%, 36%, and 46% in compressive strength, respectively. Conversely, equivalent slag replacements involved smaller impacts, with strength reductions of 6%, 19%, and 23%, respectively [20].

RHA helps lower CO_2_ emissions in concrete production by partially replacing cement, which is primarily responsible for carbon emissions due to its energy-intensive manufacturing process. RHA is made from agricultural waste, making it a sustainable and low-carbon alternative. Similarly, fly ash—a byproduct of coal burning—and ground-granulated blast-furnace slag (GGBS), a byproduct of steel production, also serve as cement replacements, aiding in emission reductions [11]. Among these, GGBS generally achieves the greatest reduction in CO_2_ emissions, followed by fly ash, due to its higher potential for cement replacement and lower embodied carbon [11]. While RHA is effective and environmentally friendly, especially in rice-producing regions, it typically yields less CO_2_ reduction compared to slag and fly ash. This is because RHA is often used in smaller replacement percentages, and its production requires controlled burning. However, when produced under ideal conditions, RHA’s higher silica content (ranging from 85% to 95%) can enhance its reactivity, allowing for higher cement replacement rates without compromising strength. This makes RHA a more efficient option for CO_2_ reduction than traditional supplementary cementitious materials [25].

The results indicated that 5–10% RHA can be used as a substitute for cement in concrete, as it shows only a minor reduction in strength at 28 days (between 9–18%), which further decreases during longer curing periods (approximately 5–8% at 90 days). Utilising RHA in concrete offers considerable environmental and economic advantages, making it a sustainable alternative to conventional cement. Since RHA is a byproduct of agriculture, its use helps mitigate the disposal issues related to rice husk waste, which is often burned or dumped in landfills, causing air and soil pollution. Incorporating RHA into concrete not only addresses these disposal problems but also reduces production costs by partially replacing cement, which is both energy-intensive and expensive to produce. Additionally, the pozzolanic properties of RHA enhance the strength and durability of concrete, leading to improved long-term cost efficiency. From an environmental standpoint, the use of RHA lowers CO_2_ emissions by reducing the demand for clinker production, a significant source of greenhouse gases in cement manufacturing. Moreover, RHA-based concrete supports sustainability by promoting circular economy principles, transforming waste into valuable construction materials while conserving natural resources. Its application fosters greener construction practices, aligning with global efforts to reduce carbon footprints and promote eco-friendly building solutions.

## 5. Data-Based Model Results

**Figure 10:**
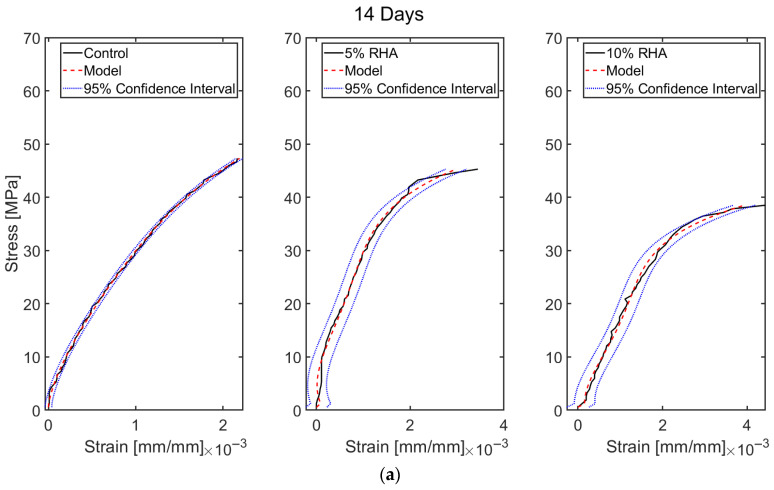

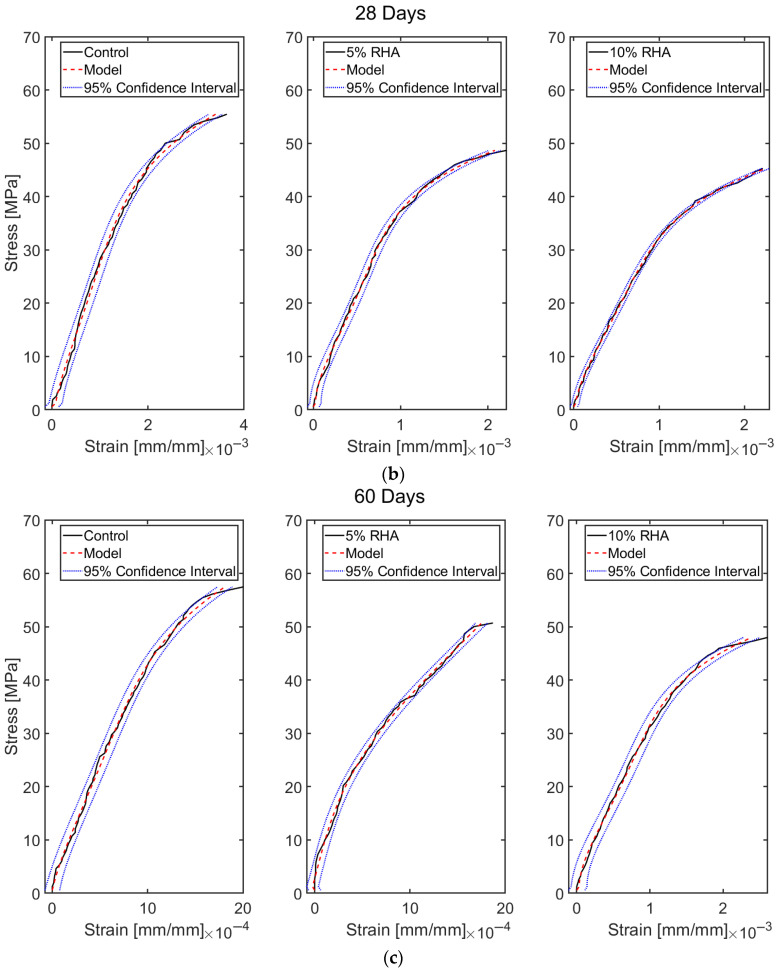

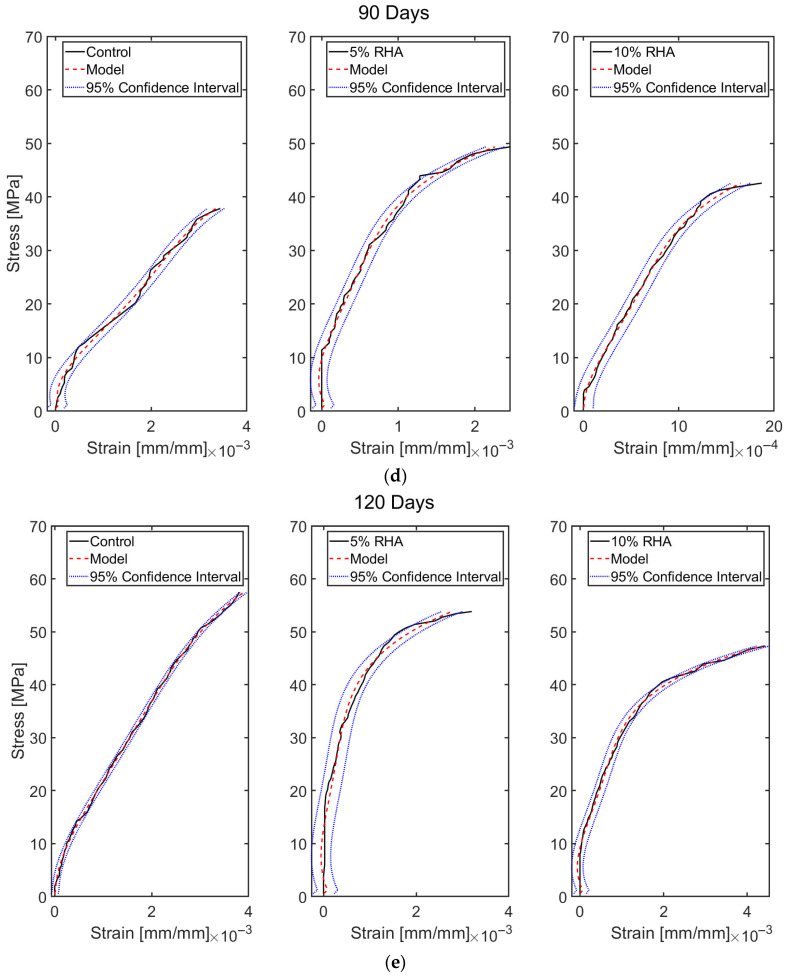
Predicted stress–strain profiles of concrete mixes with RHA compared with the experimental results and 95% confidence interval at different curing ages (14, 28, 60, 90, and 120 days).

**Figure 11:**
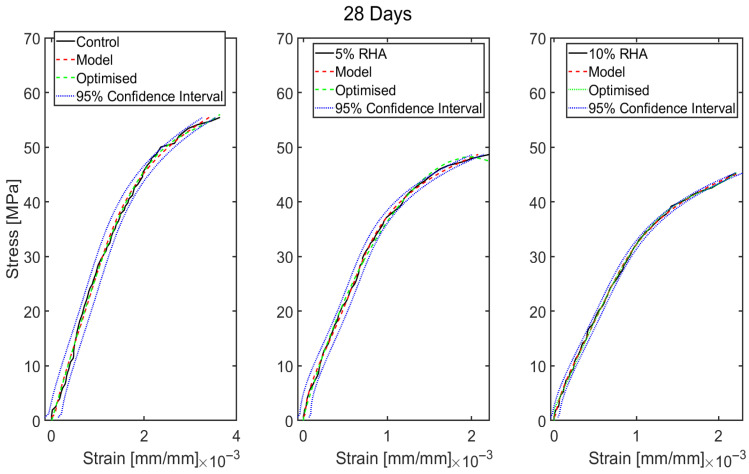
Optimised stress–strain profiles of the control and concrete with 5% and 10% RHA at 28 days curing.

**Figure 12:**
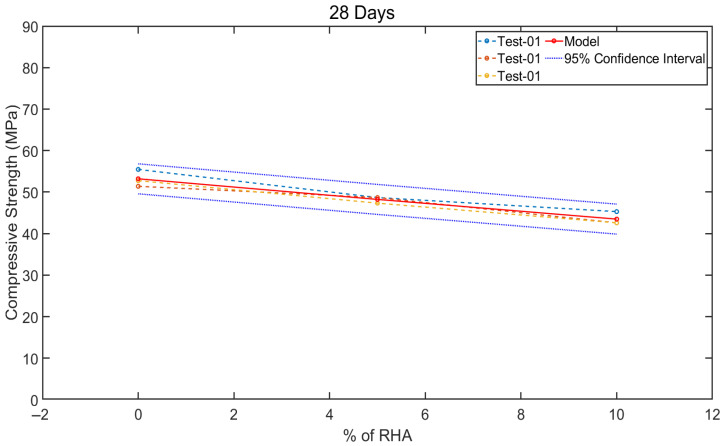
Model outcomes for the compressive strength of control (0% RHA) and concrete with 5% and 10% RHA at 28 days.

**Figure 13:**
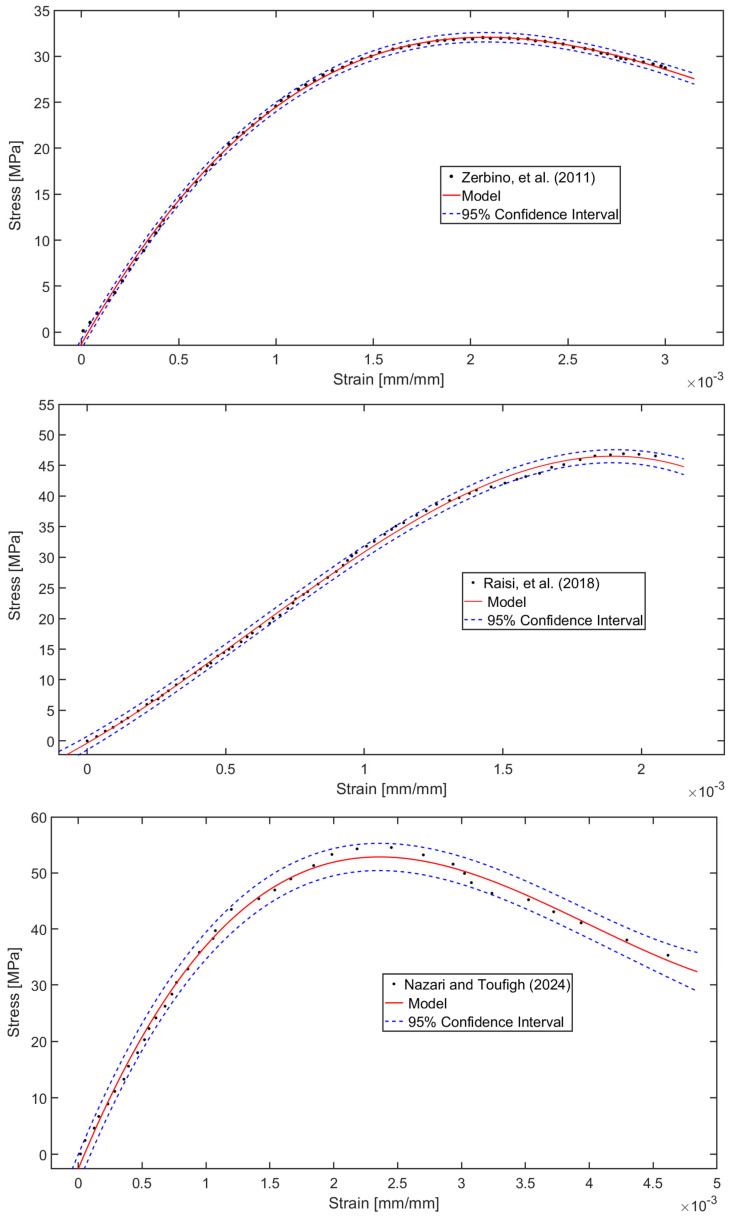
Validation of the model’s stress–strain profile prediction for concrete mixes fabricated with RHA, as reported in the literature [31,65,66].

The stress–strain profiles of concrete mixes were developed mathematically, including a control mix with 100% OPC and two mixes with 5% RHA and 10% RHA. As illustrated in Figure 6, the behaviour of some specimens was inconsistent due to premature failure. Therefore, representative stress–strain data from three specimens of each concrete mix were used for the model. The models were created for various scenarios, and their performances were compared with experimental results and a 95% confidence interval for all cases to assess the model’s performance further. The confidence levels were estimated based on the standard deviation in this study, with a 95% confidence interval (see Figure 10, Figure 11, Figure 12 and Figure 13). The cases studied can be categorised into two distinct scenarios: (i) based on curing duration (e.g., 14 days, 28 days, 60 days, 90 days, and 120 days) and (ii) based on the dosages of RHA (e.g., 0%, 5%, and 10%). The model outcomes are illustrated in Figure 10a–e. The outcomes of the models at all curing ages and for all mixes (control, 5% RHA, and 10% RHA concrete) strongly agreed with the experimental results, effectively reflecting the stress–strain profiles for all curing conditions, although some minor deviations were observed. Initially, the concrete specimens exhibited linear elastic behaviour, which the models accurately captured at various loads across different mixes and curing ages, showing a direct correlation between deformation and load. However, as the loads increased and the tensile strength of the concrete was exceeded, nonlinear effects began to appear due to cracking. This led to the propagation of cracks and the eventual failure of the concrete. While the model was able to reliably predict almost all behaviours up to the point of failure, accuracy slightly decreased after cracking occurred due to the highly nonlinear response of the material. Overall, the models performed outstandingly in all curing ages and mixes.

To justify the model’s efficacy, the coefficient of determination ($R^{2}$) values for all the models were determined, and the value for the control cases is $R^{2}=0.9993$ for 14 days, $R^{2}=0.9950$ for 28 days, $R^{2}=0.9951$ for 60 days, $R^{2}=0.9952$ for 90 days, and $R^{2}=0.9993$ for 120 days. Similar excellent $R^{2}$ values have been noticed for 5% RHA and 10% RHA cases for 14 days, 28 days, 60 days, 90 days, and 120 days. The strong alignment between data-driven computational models and experimentally obtained test results demonstrates their high predictive accuracy and robustness. This indicates their effectiveness in simulating real-world structural behaviour. The close correlation suggests that the models successfully capture the underlying physical mechanisms of concrete performance, including key factors such as material composition, load distribution, and failure modes. By consistently matching experimental outcomes across various testing scenarios—such as differing strength levels, curing ages, and varying RHA content—the models prove their reliability for practical engineering applications. Their validated performance ensures that they can be confidently used in future concrete structural analysis tasks, including assessments of load-bearing capacity, durability predictions, and optimisation of concrete formulations. Moreover, their accuracy supports their integration into design workflows, allowing engineers to reduce reliance on costly and time-consuming physical testing while maintaining high levels of safety and efficiency in concrete structure design. This validation highlights the potential of data-driven approaches to enhance innovation in construction materials and structural engineering practices.

Although the proposed models showed strong predictive accuracy in estimating the stress–strain behaviour of concrete mixtures, optimisation was carried out to explore possible improvements in their performance. This was especially true for control specimens and mixes containing 5% and 10% RHA at the standard 28-day curing period, which is the conventional benchmark for design strength evaluation. Given that the models already exhibited high precision across all curing ages, the optimisation was selectively applied to the 28-day data to streamline computational effort without compromising analytical rigour. The optimised model exhibited slight improvements, achieving even closer alignment with experimental stress–strain curves, as visually confirmed in Figure 11. A comparative assessment was carried out between the optimised results, the original model predictions, and the 95% confidence intervals to evaluate the degree of enhancement.

It is worth noting that since the initial models already performed exceptionally well, the optimisation process did not produce drastic improvements in predictive capability. This suggests that optimisation yields the most substantial benefits when baseline models struggle to accurately replicate experimental behaviour, serving as a corrective tool rather than a necessity for already high-performing systems. Nevertheless, optimisation remains a valuable strategy for researchers seeking incremental refinements or addressing specific discrepancies in model predictions. Its utility lies in fine-tuning parameters to achieve the highest possible accuracy, particularly in cases where initial model performance is suboptimal.

The 28-day compressive strength results for control concrete (0% RHA) and mixtures incorporating 5% and 10% RHA were numerically modelled and graphically represented in Figure 12. As shown in Figure 10, since the models demonstrated high accuracy at all curing ages, the compressive strength results focused solely on 28-day data to reduce computational costs while maintaining reliability. This figure compares the model’s predictions with the experimentally measured compressive strengths—derived from triplicate tests for each mix—along with their associated 95% confidence intervals, providing a robust evaluation of the model’s predictive accuracy. Only one of the three experimental test datasets was utilised to develop the model, while the remaining two were reserved for validation, ensuring an unbiased assessment of its generalisability. The comparative performance of the model, as illustrated in Figure 12, reveals a strong agreement between the predicted and experimental strength values. Notably, the model’s predictions exhibit a more linear and consistent trend compared to the experimental variability, suggesting its reliability in capturing the underlying mechanical behaviour of the concrete mixtures. These findings confirm that the model replicates the experimental outcomes with high precision and provides a stable and repeatable framework for strength prediction, which is particularly valuable for mix design optimisation and quality control in practical applications.

## 6. Data-Based Model Validation with Literature Findings

The predictive capabilities of the developed data-driven models were rigorously assessed by comparing simulated results against experimentally obtained stress–strain relationships for concrete mixtures incorporating RHA, as documented in established literature sources [31,65,66]. This validation process, graphically represented in Figure 13, involved a meticulous comparison between the model’s output and empirical data from independent studies. The data was supplemented with 95% confidence intervals to statistically quantify the reliability of the prediction. The comparative analysis revealed exceptional congruence between the computational simulations and physical test results across all evaluated mix formulations [31,65,66]. The observed $R^{2}$ values are very high, such as $R^{2}=0.9993$ for study [31], $R^{2}=0.9989$ for the study in [65], and $R^{2}=0.9957$ for [66], respectively. The model demonstrated particular proficiency in capturing key mechanical characteristics, including the elastic deformation phase, strain-hardening behaviour, and ultimate failure points, as evidenced by the close alignment with published experimental curves [31,65,66]. This robust correlation highlights the model’s ability to accurately replicate the complete stress–strain response of diverse concrete systems with varying RHA percentages. Statistical evaluation of the prediction errors confirmed consistently high accuracy levels, with mean absolute percentage errors remaining below 5% for all critical stress thresholds. While the current iteration already exhibits superior predictive performance for both conventional and RHA-modified concrete systems, ongoing research initiatives are focused on implementing advanced optimisation algorithms to refine the model’s precision further. These enhancements target specific aspects of the constitutive relationships, particularly in modelling the post-peak behaviour and damage accumulation patterns. The successful validation against multiple independent experimental datasets not only confirms the model’s immediate applicability for structural analysis but also establishes a strong foundation for future extensions to encompass broader ranges of supplementary cementitious materials and loading conditions.

## 7. Conclusions

This study examines the impact of rice husk ash (RHA) as a partial cement replacement (5% and 10% by weight) on the stress–strain response and strength characteristics of concrete over various curing periods (14, 28, 60, 90, and 120 days). A data-driven mathematical model was developed to predict these properties and subsequently validated and optimised against experimental results and existing literature. Findings indicate that incorporating RHA led to a 17% increase in slump values, attributed to the reduced temperature (12% lower) of fresh RHA-mixed concrete compared to control concrete (100% OPC-Ordinary Portland Cement). While stress–strain curves exhibited comparable trends across all mixes, those containing 5% RHA and subjected to extended curing durations demonstrated particularly consistent behaviour. Though the slope of the stress–strain curve (i.e., elastic modulus) decreased with the increased content of RHA, the slope improved at the higher curing age, especially for the concrete mix with 5% RHA compared to the control concrete. The compressive strength showed a marginal decline (9–18% at 28 days) with higher RHA content, although this reduction decreased over time (8% at 90 days). The reduction in elastic modulus and strength is primarily attributed to three mechanisms: (1) the dilution effect, where cement replacement reduces the initial clinker content, slowing early hydration; (2) incomplete pozzolanic reactivity, as RHA’s silica reacts with calcium hydroxide (Ca(OH)_2_) to form C-S-H gel only over time, delaying strength development; and (3) microstructural porosity, caused by unburnt carbon or irregular RHA particles, which disrupts packing density. While early strength may decline (e.g., 9–18% at 28 days), prolonged curing compensates for pozzolanic reactions, reducing the gap at later ages (e.g., 90+ days).

The proposed model accurately replicated the stress–strain behaviour of both control and RHA-modified mixes (5% and 10%), where the value for the control cases are $R^{2}=0.9993$ for 14 days, $R^{2}=0.9950$ for 28 days, $R^{2}=0.9951$ for 60 days, $R^{2}=0.9952$ for 90 days, and $R^{2}=0.9993$ for 120 days. Similar excellent $R^{2}$ values have been noticed for 5% RHA and 10% RHA cases for 14 days, 28 days, 60 days, 90 days, and 120 days. Furthermore, the optimised models enhance their predictive capability slightly, particularly in the post-cracking nonlinear regime. Given the high predictive accuracy of the initial models, the optimisation process yielded only marginal improvements in performance. The models also closely align with the experimental compressive strength values. Additionally, the model precisely estimated the independent stress–strain data from the literature [31,65,66], demonstrating robust performance even under pronounced nonlinear deformations. The observed $R^{2}$ values are very high, such as $R^{2}=0.9993$ for the study in [31], $R^{2}=0.9989$ in [65], and $R^{2}=0.9957$ in [66], respectively.

The present study reveals that 5–10% RHA can be used as a cement replacement to produce sustainable concrete, due to a lower strength reduction at longer curing ages (5–8% at 90 days). Using RHA as a partial cement replacement in concrete also presents a sustainable solution to multiple environmental and economic challenges. As a widely available agricultural byproduct, RHA reduces the need for landfill disposal, mitigating dumping issues while promoting a circular economy. Its incorporation lowers CO_2_ emissions by decreasing clinker demand in cement production, contributing to climate change mitigation. Additionally, RHA’s low cost compared to OPC enhances affordability, particularly in rice-producing regions. From an environmental perspective, RHA utilisation minimises air pollution caused by the open burning of rice husks, while its pozzolanic properties improve concrete durability. To maximise benefits, future efforts should focus on standardising RHA processing to ensure consistent quality, optimising replacement ratios (5–10%) to balance strength and sustainability, and promoting policy incentives for industrial adoption. This approach aligns with global sustainability goals by transforming waste into valuable construction material, reducing reliance on virgin resources, and lowering the carbon footprint of concrete.

Using RHA as a replacement for cement offers several benefits, but it also has limitations in concrete applications. One significant issue is the variability in its chemical and physical properties, which can result from different combustion conditions and husk sources. This variability can impact the consistency of its performance. Additionally, high levels of unburnt carbon can diminish the pozzolanic reactivity of RHA and increase water demand, ultimately weakening the concrete. Grinding RHA to a fine particle size for effective use is also energy-intensive, and its low bulk density presents challenges for transportation and storage. Although RHA is an environmentally friendly supplementary cementitious material, especially in regions where rice is cultivated, its contribution to CO_2_ reduction is generally less than that of slag and fly ash. This is mainly due to its limited replacement ratios in concrete mixes and the need for carefully controlled combustion during production, both of which restrict its overall carbon-saving potential. Moreover, an inconsistent supply and the absence of standardised processing methods hinder its large-scale adoption in the construction industry. Despite its pozzolanic advantages, these limitations limit the widespread use of RHA in concrete.

Future research on RHA concrete should focus on optimising combustion conditions by adjusting heating rates, burning temperatures, and cooling methods to improve the properties of the concrete. Additionally, chemical characterisation is necessary to ensure consistent pozzolanic reactivity. Comprehensive durability studies should examine long-term performance against chemical attacks, fire behaviour, carbonation, and freeze–thaw cycles. Furthermore, research should explore optimal replacement ratios and the synergistic effects of combining RHA with other supplementary cementitious materials such as slag, fly ash, and silica fume. Assessing the environmental advantages through lifecycle analysis will encourage wider industrial adoption. Ultimately, field validation through real-world structures and exposure trials is crucial for evaluating performance under diverse environmental conditions, thereby ensuring the practicality and reliability of sustainable construction.

## Figures and Tables

**Table 1:** Physical properties of NCS and NS (Note: SSD: saturated surface dry).

Properties	Unit	NCS	NS
Specific gravity (SSD)	-	2.73	2.54
Unit weight (SSD)	kg/^3^	1550	1530
Absorption capacity	%	1.00	3.10
Fineness modulus	-	7.43	2.82

**Table 2:** Chemical composition of OPC, RHA, NCS, and NS.

Chemical Composition	OPC (%)	RHA (%)	NCS (%)	NS (%)
Al_2_O_3_	5.03	0.30	11.81	4.55
SiO_2_	21.47	91.3	50.32	84.56
CaO	65.01	0.21	12.32	0.85
Fe_2_O_3_	3.76	0.12	13.44	4.15
SO_3_	2.04	0.24	0.15	-
MgO	0.92	0.31	5.53	0.37
K_2_O	-	2.65	0.69	4.18
TiO_2_	-	0.15	2.02	0.31
P_2_O_5_	-	-	0.29	0.13
Na_2_O	-	1.52	3.11	0.82
MnO	-	-	0.17	0.04
ZrO_2_	-	-	-	-
SrO	-	-	0.06	0.02
Cr_2_O_3_	-	-	0.07	-

**Table 3:** Mix design of concrete mixes (kg/m^3^).

Mix ID	OPC	RHA	NCS	NS	Water
Control	500	0	974	745	175
5% RHA	475	25	974	745	175
10% RHA	450	50	974	745	175

## Data Availability

The original contributions presented in this study are included in the article. Further inquiries can be directed to the corresponding author.
